# Introducing Newborn Screening for Severe Combined Immunodeficiency (SCID) in the Dutch Neonatal Screening Program

**DOI:** 10.3390/ijns4040040

**Published:** 2018-12-12

**Authors:** Maartje Blom, Robbert G.M. Bredius, Gert Weijman, Eugènie H.B.M. Dekkers, Evelien A. Kemper, M. Elske van den Akker-van Marle, Catharina P.B. van der Ploeg, Mirjam van der Burg, Peter C.J.I. Schielen

**Affiliations:** 1 Department of Pediatrics, Laboratory for Immunology, Leiden University Medical Center (LUMC), P.O. Box 9600, 2300 RC Leiden, The Netherlands; 2 Department of Biologicals, Screening and Innovation, Centre for Health Protection, National Institute of Public Health and the Environment (RIVM), P.O. Box 1, 3720 BA Bilthoven, The Netherlands; 3 Department of Pediatrics, Leiden University Medical Center (LUMC), P.O. Box 9600, 2300 RC Leiden, The Netherlands; 4 Department of Vaccine Supply and Prevention Programmes, National Institute for Public Health and the Environment (RIVM), P.O. Box 1, 3720 BA Bilthoven, The Netherlands; 5 Centre for Population Screening, National Institute of Public Health and the Environment (RIVM), P.O. Box 1, 3720 BA Bilthoven, The Netherlands; 6 Department of Clinical Chemistry, IJsselland Hospital, P.O. Box 690, 2900 AR Capelle aan den IJssel, The Netherlands; 7 Department of Biomedical Data Sciences, section Medical Decision Making, Leiden University Medical Center (LUMC), P.O. Box 9600, 2300 RC Leiden, The Netherlands; 8 Department of Child Health, TNO, P.O. Box 3005, 2301 DA Leiden, The Netherlands

**Keywords:** severe combined immunodeficiency, SCID, newborn screening, T-cell receptor excision circles, TREC, SONNET study

## Abstract

The implementation of newborn screening for severe combined immunodeficiency (SCID) in the Netherlands is a multifaceted process in which several parties are involved. The Dutch Ministry of Health adopted the advice of the Dutch Health Council to include SCID in the Dutch newborn screening program in 2015. As newborn screening for SCID is executed with a new, relatively expensive assay for the Dutch screening laboratory, an implementation pilot study is deemed instrumental for successful implementation. A feasibility study was performed in which the practicalities and preconditions of expanding the newborn screening program were defined. Cost-effectiveness analysis (CEA) indicated that SCID screening in the Netherlands might be cost-effective, recognizing that there are still many uncertainties in the variables underlying the CEA. Data and experience of the pilot study should provide better estimates of these parameters, thus enabling the actualization of CEA results. Prior to the implementation pilot study, a comparison study of two commercially available SCID screening assays was performed. A prospective implementation pilot study or so-called SONNET study (SCID screening research in the Netherlands with TRECs) started in April 2018 and allows the screening for SCID of all newborns in three provinces of the Netherlands for one year. Based on the results of the SONNET study, the Dutch Ministry of Health will make a final decision about national implementation of newborn screening for SCID in the Netherlands.

## 1. Introduction

**Figure 1:**
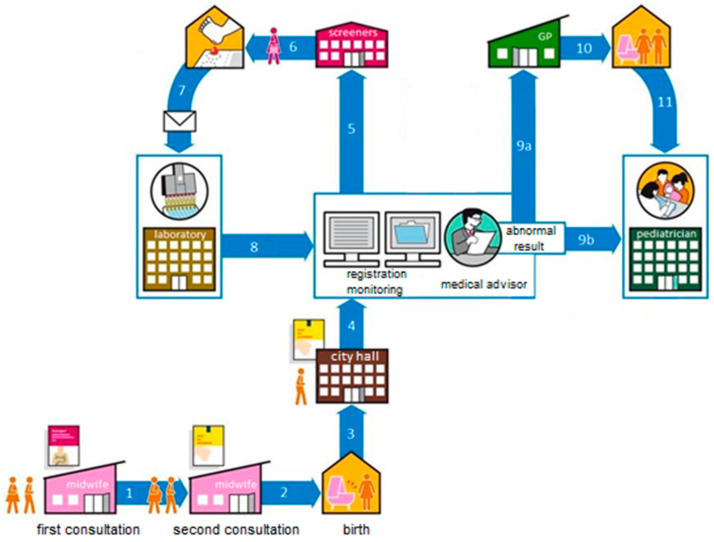
The primary process of the Dutch newborn screening program. Expecting parents will receive information about the newborn screening program during the first [1] and second consultation with the midwife [2]. The first information brochure briefly mentions newborn screening, whereas the second brochure elaborates on the objectives, disorders and newborn screening process. During registration of a newborn at city hall, the second information brochure will be handed out as well (3). The screening organisations (5) will be informed about the registration of the newborn at city hall (4), after which screeners will visit the parents at home or in the hospital (6). They will perform the heel prick and send the heel prick card by post to one of the five screening laboratories (7). The heel prick cards are then analysed and the results are registered in the laboratory information system (LIMS) and the national monitoring database Praeventis (8). Abnormal results are forwarded to the general practitioner (GP) (9a) and paediatrician (9b) by the medical advisor. Medical advisors coordinate logistics of the referral procedure. GPs will visit the parents and their newborn (10) and inform them about the referral of their newborn to the paediatrician within the pre-set referral time (11).

This article provides a brief overview of the developments in the Netherlands with regard to newborn screening for severe combined immunodeficiency (SCID) for the special themed “Newborn Screening for primary immunodeficiency diseases—Past, Present and Future” issue. The Dutch newborn screening program started in 1974 with screening for phenylketonuria (PKU). Since then, the number of disorders in the newborn screening program expanded significantly and newborns in the Netherlands are now being screened for nineteen disorders. Each year approximately 175,000 newborn blood spot screening tests are performed. Participation in the newborn screening program has remained stable over time, and was approximately 99.2% in 2016 [1]. Newborn blood spot collection is carried out as soon as possible within 72 to 168 h after birth. Newborn screening analyses are performed in one of the five screening laboratories in the Netherlands. The screening laboratory of the National Institute of Public Health and the Environment (RIVM) serves as a reference laboratory. The primary process of newborn screening in the Netherlands is depicted in Figure 1. At the national level, the screening program is organised by the RIVM’s Centre for Population Screening (CvB) on behalf of the Dutch Ministry of Health, Welfare and Sport. The Programme Committee for Newborn Blood Spot Screening, which was established by the RIVM CvB, advises the RIVM with regard to the program’s national coordination. Neonatal screening is dynamic and subject to change. Treatment options and screening test methods for certain disorders have improved significantly over the past years [2,3]. The development of a detection method for SCID [4,5] and the implementation of this test method in the newborn screening programs of the United States [6] and other countries [7,8] raised public and expert attention to study the implementation of newborn screening for SCID in the Dutch newborn screening program.

## 2. Newborn Screening Recommendations by the Health Council of the Netherlands

The Health Council of the Netherlands, established in 1902, is an independent scientific advisory body whose remit it is to advise the government and Parliament with respect to public health issues and health (care) research. Previous reports of the Health Council in 2005 and 2010 resulted in the expansion of the newborn screening program to seventeen disorders [9,10]. In 2012, the Minister for Health, Welfare and Sport asked the Council for a new advisory report on newborn screening that would mainly focus on the recommendation of new disorders to be implemented in the newborn screening program. Other issues that also were requested to be addressed were the criteria for inclusion of disorders in neonatal screening, conditions currently eligible for inclusion in screening, and how incidental secondary findings should be dealt with in the program. The report ‘Neonatal screening: new recommendations’ came out in 2015, in which the Committee recommended to add fourteen new conditions to the neonatal screening program. These new conditions are alpha- and beta-thalassemia, carnitine acylcarnitine translocase deficiency (CACT), carnitine palmitoyltransferase deficiency type 1 (CPT1), carnitine palmitoyltransferase deficiency type 2 (CPT2), galactokinase deficiency (GALK), guanidinoacetate methyltransferase deficiency (GAMT), beta-ketothiolase deficiency (BKT), methylmalonic acidemia (MMA), mucopolysaccharidosis type 1 (MPS I), organic cation transporter 2 deficiency (OCTN 2), propionic acidemia (PA), X-linked adrenoleukodystrophy (X-ALD) and SCID [11]. The report also included advice about X-linked a-gammaglobulinemia (XLA), stating that a research study of the test characteristics of the kappa-deleting recombination excision circles (KREC) test should be initiated before inclusion of XLA in the neonatal screening program can be reconsidered. The Committee stated that newborn screening for SCID would prevent significant, irreversible damage and yield substantial health gains for the affected child. Although the detection of T-cell receptor excision circles (TRECs) by PCR is more complicated and expensive than other neonatal test methods, it would seem to stay within acceptable limits of efficacy. The Committee did recommend a more extensive pilot study and an exact cost-benefit analysis as part of the implementation process.

## 3. The Response of the Dutch Ministry of Health, Welfare and Sport

On 9 July 2015, the Dutch Ministry of Health, Welfare and Sport published a policy position paper entitled “Newborn blood spot screening”. In this briefing, the Minister adopted the advice of the Health Council to extend the newborn blood spot screening with fourteen new disorders. The Minister subdivided the implementation process of these disorders into three phases, involving short, medium and long preparation times. Although other countries have already screened for SCID for many years [6], TREC detection based on PCR is a relatively expensive test method, which has yet to be validated in the Dutch screening laboratory. A thorough process of implementation of newborn screening for SCID would therefore require a long preparation time. The Minister did, however, urge to give priority to the implementation of SCID screening, as SCID would be the first disorder in the newborn screening program that could not only be treated but completely cured. The Minister also asked the Centre for Population Screening to carry out a feasibility study to determine the practicalities involved with the implementation of fourteen new disorders [12].

## 4. Feasibility Study by the Centre for Population Screening

The Centre for Population Screening of the RIVM carried out a feasibility study commissioned by the Ministry of Health to investigate the practicalities and preconditions of expanding the newborn screening program. The expansion is a complex process due to the large number of disorders, changes in logistics and organisation of screening laboratories, availability and quality of test methods and new follow-up procedures. The feasibility study report was published in July 2017 and stated that implementation of the fourteen new disorders would only be feasible in a phased manner and if the following conditions are met: adequate staffing levels and financial means, the availability of flexible IT amenities, and a good interface with the health services [13]. The report emphasised the importance of second tier or even third tier testing and post-analytic tools to prevent large numbers of false positive referrals. It is of great importance that the present neonatal screening program is not affected by the planned expansion and its associated preparations. At the end of each preparatory phase, the Secretary of State for Health, Welfare and Sport must decide whether the condition can enter the implementation phase or whether further research is required. Alpha- and beta-thalassemia were already implemented in the newborn screening program in the beginning of 2017. The proposed planning of implementing the remaining disorders follows a five-year plan. Implementation of CPT1, MMA and PA is deemed feasible by the end of 2019. Other disorders will follow by the end of 2020 (MPS I and GALK), by the end of 2021 (CACT, CPT, BKT, OCTN2, SCID and X-ALD) and finally by the end of 2022 (GAMT). The total costs of expanding the newborn screening program are estimated at 14 million euros over the five-year period. The Centre for Population Screening dedicated a separate chapter to the implementation of SCID screening, as the pilot study for SCID screening requires new equipment, adjustments in the screening laboratory, training of staff, changes in the laboratory information system (LIMS) and the monitoring database Praeventis, and new referral and follow-up protocols. Parents should be informed about the SCID screening pilot during their pregnancy and after birth. This means that new brochures and leaflets with comprehensible information for parents had to be developed that fitted in to the existing information framework of the newborn screening program. Information material for professionals and health care providers about SCID and the SCID screening pilot had to be developed as well. Parents had to be formally asked for their consent for the participation of their child in the SCID screening pilot by screeners. As the Centre for Population Screening monitors outcomes of the routine screening program, the close collaboration between the pilot study project group and CvB ensures the concurrent execution of the pilot study and the routine screening program. 

## 5. Cost-Effectiveness Analysis of Newborn Screening for SCID

Cost-effectiveness studies for newborn screening for SCID have already been performed in the United States and New Zealand [14,15,16]. However, as costs and benefits of screening and treatment are likely to differ between countries and especially between continents, a cost-effectiveness analysis (CEA) for SCID was carried out by the Netherlands Organisation for applied scientific research (TNO) in collaboration with Leiden University Medical Centre (LUMC). Lifetime costs and effects of newborn screening for SCID were compared with a situation without screening in the Netherlands in a decision analysis model. Model parameters were based on literature and expert opinions, after which sensitivity analyses were performed. The results lead to the publication of a report entitled “Cost-effectiveness and cost–benefit analysis (CEA/CBA) for SCID screening within the Dutch newborn screening program” in April 2017 [17,18]. The SCID screening situation lead to additional costs for laboratory testing and follow-up diagnostics, but the costs of treatment of SCID patients were expected to decrease if newborn screening for SCID would be implemented. Although more patients would receive treatment in the form of hematopoietic stem cell transplantation, the early detection of the disease would result in lower transplantation-associated costs [19]. The long-term treatment costs would be lower as well, as early transplantation results in more favourable health outcomes. The results for the Netherlands are comparable with cost-effectiveness studies in the United States [14,15] and indicate that SCID screening might be cost-effective, but the range of possible cost-effectiveness ratios is broad due to many parameter-associated uncertainties such as the incidence of SCID, costs of screening tests and costs of late transplantation. In conclusion, SCID screening in the Netherlands could be cost-effective but, due to many uncertainties, an extensive pilot study should be performed to help actualise the results of this CEA.

## 6. SCID Screening Assays Comparison Study

**Figure 2:**
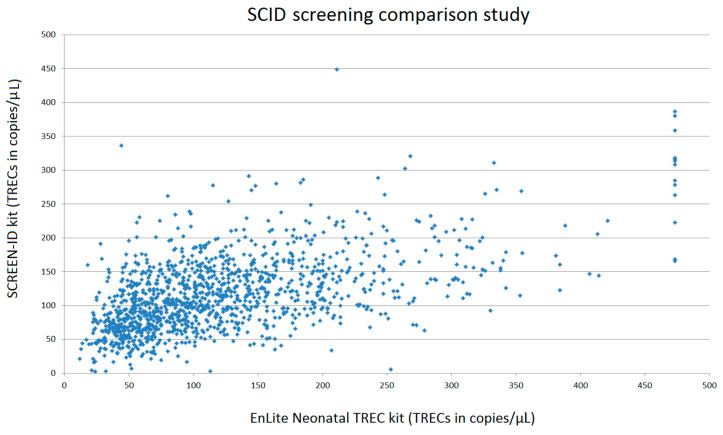
Comparison of TREC-levels in 1272 heel prick cards analysed with both SCID screening assays. Data in the diagram are displayed in a scatter-plot with EnLite Neonatal TREC analyses on the x-axis and SCREEN-ID analyses on the y-axis.

Prior to the prospective pilot study, a small-scale pilot study had already been performed in the Netherlands to show that the TREC assay is a suitable method for the Dutch newborn screening situation [20]. Since then, other newborn screening assays for SCID became commercially available and in order to select the most suitable assay for the large-scale prospective pilot study, an objective comparison study was performed. There were two commercially available newborn SCID screening assays at the time of the comparison study: the EnLite™. Neonatal TREC kit of PerkinElmer and the SCREEN-ID neonatal screening kit of ImmunoIVD (now called SPOT-it™ neonatal screening assay). Based on pre-set objective comparison criteria (established and approved before the evaluation phase by several parties including the CvB), the test qualities of both available SCID screening assays and their applicability for the Dutch screening situation were evaluated. The EnLite Neonatal TREC assay is a dried blood spot assay employing PCR-based nucleic acid amplification and time-resolved fluorescence resonance energy transfer (TR-FRET) technology. The EnLite Neonatal TREC assay involves punching of dried blood spot specimens with a 1.5 mm punch head, adding elution buffer and starting elution incubation. After elution, reagent mixture is added and thermal incubation consisting of DNA amplification and probe hybridisation is carried out. Signals from hybridised TREC and β-actin (internal control for monitoring amplification) probes are measured with the VICTOR™ EnLite Instrument (PerkinElmer). The assay is an in vitro diagnostic device intended for the semi-quantitative, multiplex determination of TREC and β-actin [21]. The SCREEN-ID assay is an in vitro diagnostic kit intended for routine screening of fresh, prospective neonatal dried blood spot samples. The assay is an all-in-one system for the quantitation of T-cell specific TRECs and/or B-cell specific KRECs as well as quality control marker β-actin using quantitative multiplex real-time PCR (qPCR). The SCREEN-ID assay employs regular 3.2 mm Guthrie card spots and features a Filter plate concept to rinse samples prior to analysis. The kit includes all necessary reagents pre-arranged in a set of Elution and qPCR plates and requires only two pipetting steps. As the triplex-detection chemistry for TRECs, KRECs and β-actin markers is independent of each analyte, the user can tailor the required diagnostic output of the SCREEN-ID assay. Some users might limit the screening approach to TRECs only, while others choose to report both TRECs and KRECs [22]. As the Dutch Health Council and Ministry of Health decided that newborn screening for SCID should be solely based on TREC detection, only the TRECs—and not the KRECs—detection feature of the assays were used and evaluated in this comparison study. To compare both SCID screening assays, 1272 anonymised fresh heel prick samples from the Dutch newborn screening program were analysed. Moreover, peripheral blood from eight patients with a clinical, genetically confirmed SCID diagnosis (affected genes: RAG1, *n* = 3; RAG2, *n* = 2; IL2Rg, *n* = 1; *XLF n* = 1; Artemis *n* = 1) were included as well. Both assays were performed adhering strictly to the instructions of the manufacturer with the recommended instruments. Both manufacturers provided the researcher with personal training before performing the analyses. There were no deviations from either one of the protocols. The mean TREC level of 1272 anonymised Dutch heel prick cards was 123 copies/μL blood (median TREC: 102 copies/μL) for the EnLite Neonatal TREC assay and 116 copies/μL blood (median TREC: 109 copies/μL) for the SCREEN-ID assay (see Table 1). The number of heel prick cards below the 2.5 percentile-mark was identical for both assays (*n* = 32). However, of these 32 heel prick cards, only eight cards presented with TREC levels below the 2.5 percentile in both assays. The remaining 24 heel prick cards showed disparate TREC levels due to poor amplification and low β-actin levels in either one of the assays. In the routine screening program, these samples would require retesting in duplicate. Retesting was not performed during the comparison study, as retest rates and referral rates based on this small sample size would not be reliable comparison criteria. In this study, an experimental TREC cut-off level of 30 copies/μL was used for the EnLite Neonatal TREC assay to distinguish screen positive samples, based on advisory information of the manufacturer (PerkinElmer). For the SCREEN-ID kit an experimental TREC cut-off level of 6 copies/μl was used, based on advisory information of the manufacturer (ImmunoIVD). Both manufacturers recommend performing a large sample size pilot study to establish a preferred cut-off value based on the normal population distribution. With the TREC cut-off set at 30 copies/μL blood, 38 samples (3.0%) required a retest after the initial analysis with the EnLite Neonatal TREC assay. With the TREC cut-off set at 6 copies/μL blood, 5 samples (0.39%) required a retest after the initial analysis with the SCREEN-ID assay. As mentioned above, the number of samples below the cut-off value was not included as a comparison criterion, as each laboratory should establish a cut-off value based on a large sample size pilot study. The distribution of TREC levels in the analysed heel prick cards is displayed in Figure 2. Samples of all eight genetically confirmed SCID patients had absent TREC levels, below the cut-off levels proposed in the respective kit-inserts. 

## 7. SCID Screening Prospective Implementation Pilot Study

**Figure 3:**
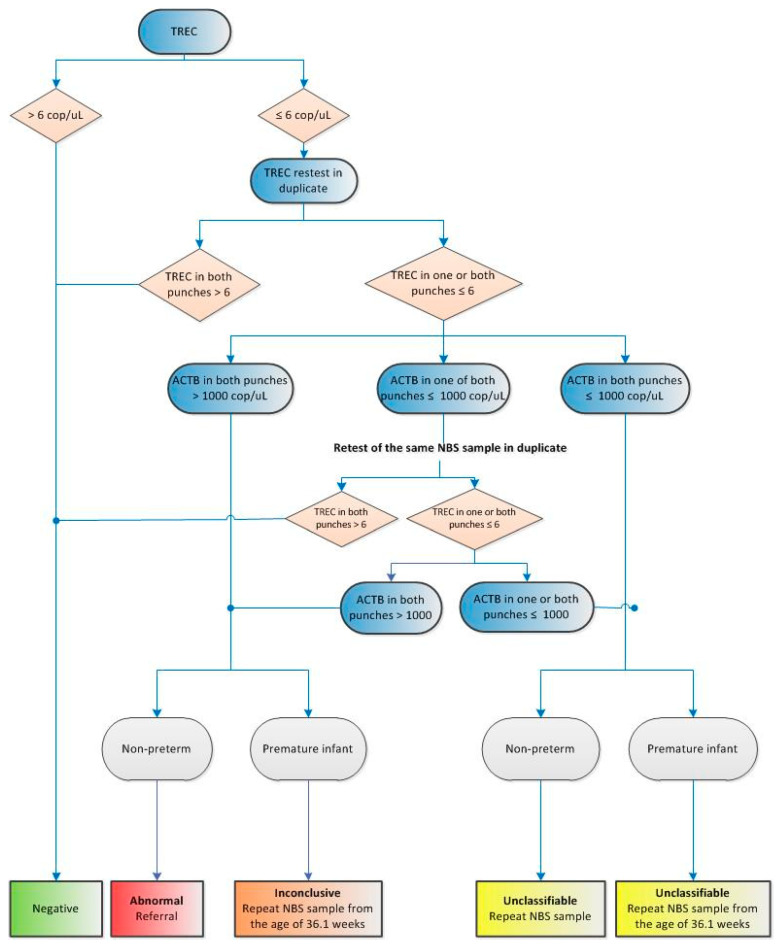
A flow chart of the TREC-assay and the referral procedure. Premature infants are newborns with gestational age ≤36 weeks and birth weight ≤2500 g. TREC are T-cell receptor excision circles and *ACTB* is β-actin, the internal reference control.

As previously described, implementation of neonatal screening for SCID is complex due to expensive screening methods and intensive treatment options, such as hematopoietic stem cell transplantation. Moreover, there are a number of uncertainties, ranging from the expected number of referrals and analytical difficulties to unanticipated logistic challenges and unexpected screening outcomes. These uncertainties might seriously hamper the introduction of SCID screening in the routine program. In order to enable a flawless implementation of SCID screening, a prospective implementation pilot study within the routine screening programme supported by The Netherlands Organisation for Health Research and Development (ZonMw) is being executed. ZonMw funds health research and promotes the use of the knowledge this research produces. The pilot study aims to gather knowledge about the practical implications of newborn screening for SCID, the cost-effectiveness, diagnostic and clinical follow-up issues, and the perspectives of health care providers and parents. This study will also assess the incidental findings accompanied by newborn screening for SCID, such as secondary T-lymphopenia due to congenital anomalies, or syndromes with T-cell impairment such as DiGeorge syndrome, trisomy 21, trisomy 18, and CHARGE syndrome [6]. As the Dutch Health Council has already deemed SCID a suitable candidate for the Dutch newborn screening program, that meets the Wilson and Jungner criteria [23], this pilot study does not focus on whether the TREC assay is a suitable method for the detection of SCID. The effectiveness of newborn screening for SCID has already been proven in other screening programs abroad [6,7], and previous research has provided us with a clear overview of the SCID disease in the Netherlands [24]. The implementation pilot will answer four main research questions: How can the TREC-screening method be implemented in the current neonatal screening program? What are the test qualities of TREC detection in “real life” in the Netherlands? What are the costs for introduction of SCID screening in the neonatal screening program? How can adequate information and counselling facilitate an acceptable screening process for parents and their health care? The implementation pilot or SONNET study (SCID screening Research/Onderzoek in the Netherlands with TRECs) uses the infrastructure of the Dutch newborn screening program. The TREC assay is performed in two screening laboratories (RIVM in Bilthoven and IJsselland Hospital in Capelle aan den IJssel) and includes the newborns of three provinces (Utrecht, Gelderland and Zuid Holland). The pilot study started in April 2018 and includes the yearly workload of two screening laboratories, approximately 70,000 newborns. The project plan is based on four work packages that will be carried out over a two-year period. The first work package includes all preparatory steps required for the test phase that started on 1 April 2018. Information brochures for parents and health care providers have been distributed, informing them about SCID and the SCID screening pilot study. Parents receive information at different points in time allowing them to make an informed decision to participate in the pilot study. The information brochure contains information about the condition SCID, the goal and necessity of the implementation pilot, the advantages and disadvantages of participating, test results and privacy. Summaries of the brochure are available in English, Polish, Turkish and Arabic. Moreover, a website with additional information about the pilot and the latest development has been developed (www.sonnetstudie.nl). Informative meetings have been organised for screeners, midwifes, paediatricians and other parties involved in the pilot study. The screening laboratories have been adjusted and equipped for the PCR test method and technicians have received training from the manufacturer of the TREC assay. ICT software has been updated, enabling the SCID screening results to be included in the routine screening databases. A flow chart for the screening laboratories has been designed (see Figure 3), in which a distinction is made between full term and preterm infants (gestational age ≤36 weeks and birth weight ≤2500 g). Full term infants with an abnormal TREC result are referred to a paediatrician immunologist in one of the academic medical centres within 72 h. Immunophenotyping by flow cytometry is the first step in the diagnostic follow-up. If T-lymphocytes are absent, genetic analysis (whole exome sequencing) with a pre-set SCID gene panel will be a secondary step performed by the academic medical centre of referral. Newborns with absent T-cells will simultaneously start with the hematopoietic stem cell transplantation work-up. If T-cells are low or non-functional, extra immunological diagnostics will be carried out in addition to the SCID gene panel analysis. Even though some of the incidental secondary findings of SCID screening are incurable, protective measures and prophylaxis could still provide a health gain for the individual. The follow-up procedure for SCID and non-SCID patients has been uniformly determined by paediatricians of all participating academic medical centres. The second work package focusses on the “real-life” SCID screening phase. TRECs are measured in approximately 70,000 newborns over a period of one year, with an interim evaluation after three months of screening. During the pilot study, KRECs are measured as well, but data are anonymised and used for research purposes only. In the third work package, the CEA of 2017 will be refined with new input data obtained from the prospective pilot study. The final work package focuses on the ethical, legal, and societal implications (ELSI) of newborn screening for SCID and the expansion of the newborn screening program. SCID is a case example of a disorder with high impact secondary findings and potential false positive rates, and the perspectives of parents and their health care providers are of great value. After the large scale pilot study, the Dutch Ministry of Health, Welfare and Sport will assess the evaluation report and make a final decision about the implementation of newborn screening for SCID in the Netherlands.

## 8. International Collaboration

At the end of 2017, the RIVM organised a first meeting about SCID screening with newborn screening experts from the Karolinksa Institutet (Stockholm, Sweden) and experts of the newborn screening program of the United Kingdom. In this meeting, new developments were discussed and experiences and results were shared, providing new inspiration for all parties involved. At the 11^th^ European regional meeting of the International Society of Newborn Screening (ISNS) in Bratislava, Slovakia (October 2018), a session about newborn screening for SCID was organised in which experiences of several countries with regard to SCID screening were shared. Moreover, future developments in the field of newborn screening for primary immunodeficiencies, such as newborn screening for XLA, congenital neutropenia and IPEX syndrome were also discussed. As newborn screening for SCID in Europe is still in its infancy, with many European countries planning pilot studies or awaiting governmental implementation decisions, the RIVM is open for collaboration with all interested parties. Newborn screening for SCID might provide the perfect opportunity to initiate more international collaboration in the field of newborn screening.

## Figures and Tables

**Table 1:** Results of the analysis of 1272 fresh heel prick cards. The average, median and 2.5 percentile of both severe combined immunodeficiency (SCID) screening assays are depicted in copies/µL blood. The number of heel prick cards with T-cell receptor excision circle (TREC) levels below the 2.5 percentile and the number below the cut-off of the manufacturer are also shown.

	EnLite Neonatal TREC Assay	SCREEN-ID Assay
Average (TREC copies/μL)	123	116
Median (TREC copies/μL)	102	109
2.5 percentile (TREC copies/μL)	28	33
Number of heel prick cards below 2.5 percentile	32	32
Number of heel prick cards below manufacturer’s cut-off	38	5

The comparison criteria were subdivided into categories, each with a maximum amount of points to be awarded, namely Applicability (50 points), Analytical Procedure (205 points), Equipment and Software (65 points), Pricing (85 points) and Quality and Service (110 points). As the comparison criteria might be used for future tender procedures, the document cannot be made publically available. Both SCID screening assays turned out to be suitable TREC detecting assays for the Dutch screening laboratories. Subtle differences lead to the selection of the assay with the most awarded overall points, namely the SCREEN-ID neonatal screening assay of ImmunoIVD. The SCREEN-ID assay is therefore used in the large scale implementation pilot study.

## References

[1] van der Ploeg C.P.B., Wins S., Olthof R., Eekhout I., Verkerk P.H. (2018). The Newborn Blood Spot Screening in the Netherlands—Monitor 2017.

[2] Almannai M., Marom R., Sutton V.R. (2016). Newborn screening: A review of history, recent advancements, and future perspectives in the era of next generation sequencing. Curr. Opin. Pediatr..

[3] El-Hattab A.W., Almannai M., Sutton V.R. (2018). Newborn screening: History, Current Status, and Future Directions. Pediatr. Clin. N. Am..

[4] Baker M.W., Grossman W.J., Laessig R.H., Hoffman G.L., Brokopp C.D., Kurtycz D.F., Cogley M.F., Litsheim T.J., Katcher M.L., Routes J.M. (2009). Development of a routine newborn screening protocol for severe combined immunodeficiency. J. Allergy Clin. Immunol..

[5] Gerstel-Thompson J.L., Wilkey J.F., Baptiste J.C., Navas J.S., Pai S.Y., Pass K.A., Eaton R.B., Comeau A.M. (2010). High-throughput multiplexed T-cell-receptor excision circle quantitative PCR assay with internal controls for detection of severe combined immunodeficiency in population-based newborn screening. Clin Chem..

[6] Kwan A., Abraham R.S., Currier R., Brower A., Andruszewski K., Abbott J.K., Baker M., Ballow M., Bartoshesky L.E., Bonagura V.R. (2014). Newborn screening for severe combined immunodeficiency in 11 screening programs in the United States. JAMA.

[7] Rechavi E., Lev A., Simon A.J., Stauber T., Daas S., Saraf-Levy T., Broides A., Nahum A., Marcus N., Hanna S. (2017). First year of Israeli Newborn Screening for Severe Combined Immunodeficiency-Clinical Achievements and Insights. Front. Immunol..

[8] Chien Y., Yu H., Lee N., Ho H., Kao S., Lu M., Jaing T., Lee W., Chang K., Shieh C. (2017). Newborn Screening for Severe Combined Immunodeficiency in Taiwan. Int. J. Neonatal. Screen..

[9] Health Council of the Netherlands (2005). Neonatal Screening.

[10] Health Council of the Netherlands (2010). Neonatal Screening for Cystic Fibrosis.

[11] Health Council of the Netherlands (2015). Neonatal Screening: New Recommendations.

[12] Letter of the Dutch Minister for Health (mw. drs. E.I. Schippers) to the Chairman of the House of Representatives of the Netherlands on the Expansion of the Newborn Screening Program. http://artsenjgz.nl/nieuwsbericht/reactie-minister-van-vws-op-advies-gezondheidsraad-over-14-nieuwe-aandoeningen-in-de-hielprikscreening/>.

[13] Dekkers E.H.B.M., Klein A.W., Lock A.J.J., Vermeulen H.M. (2017). Uitvoeringstoets uitbreiding neonatale hielprikscreening. Rijksinstituut voor Volksgezondheid en Milieu.

[14] Ding Y., Thompson J.D., Kobrynski L., Ojodu J., Zarbalian G., Grosse S.D. (2016). Cost-Effectiveness/Cost-Benefit Analysis of Newborn Screening for Severe Combined Immune Deficiency in Washington State. J. Pediatr..

[15] Chan K., Davis J., Pai S.Y., Bonilla F.A., Puck J.M., Apkon M. (2011). A Markov model to analyze costeffectiveness of screening for severe combined immunodeficiency (SCID). Mol. Genet. Metab..

[16] Health Partners Consulting Group Cost-Effectiveness of Newborn Screening for Severe Combined Immune Deficiency. A Report Prepared for the National Screening Unit. https://www.nsu.govt.nz/system/files/resources/cost-effectiveness-newborn-screening-severe-combined-immune-deficiency.pdf.

[17] van der Ploeg C.P.B., van den Akker-van Marle E., Bredius R.G.M., Staal F., van den Burg M., Verkerk P. (2017). Kosteneffectiviteits- en Kostenbatenanalyse (KEA/KBA) Voor Het screenen op SCID Binnen de Nederlandse Hielprikscreening.

[18] Van der Ploeg C.P.B., Blom M., Bredius R.G.M., van der Burg M., Schielen P.C.J.I., Verkerk P.H., van den Akker-van Marle M.E. (2018). Cost-effectiveness of newborn screening for severe combined immunodeficiency. Eur. J. Pediatr..

[19] Clément M.C., Mahlaoui N., Mignot C., le Bihan C., Rabetrano H., Hoang L., Neven B., Moshous D., Cavazzana M., Blanche S. (2015). Systematic neonatal screening for severe combined immunodeficiency and severe T-cell lymphopenia: Analysis of cost-effectiveness based on French real field data. J. Allergy Clin. Immunol..

[20] Blom M., Pico-Knijnenburg I., Sijne-van Veen M., Boelen A., Bredius R.G.M., van der Brug M., Schielen P.J.C.I. (2017). An evaluation of the TREC assay with regard to the integration of SCID screening into the Dutch newbon screening program. Clin. Immunol..

[21] PerkinElmer EnLite Neonatal TREC Kit. https://newbornscreening.perkinelmer.com/products/enlite_neonatal_trec_instrument/enlite_neonatal_trec_kit.

[22] ImmunoIVD SCREEN-ID Neonatal Screening Kit. http://immunoivd.com/technology.html.

[23] Wilson J.M.G., Jungner G. (1968). Principles and Practice of Screening for Disease.

[24] De Pagter A.P.J., Bredius R.G.M., Kuijpers T.W., Tramper J., van der Burg M., van Montfrans J., Driessen G.J. (2015). Overview of 15-year severe combined immunodeficiency in the Netherlands: Towards newborn blood spot screening. Eur. J. Pediatr..

